# Orthopedic Surgery Triggers Attention Deficits in a Delirium-Like Mouse Model

**DOI:** 10.3389/fimmu.2019.02675

**Published:** 2019-11-19

**Authors:** Ravikanth Velagapudi, Saraswathi Subramaniyan, Chao Xiong, Fiona Porkka, Ramona M. Rodriguiz, William C. Wetsel, Niccolò Terrando

**Affiliations:** ^1^Department of Anesthesiology, Center for Translational Pain Medicine, Duke University Medical Center, Durham, NC, United States; ^2^Department of Psychiatry and Behavioral Sciences, Mouse Behavioral and Neuroendocrine Analysis Core Facility, Duke University Medical Center, Durham, NC, United States; ^3^Departments of Neurobiology and Cell Biology, Duke University Medical Center, Durham, NC, United States

**Keywords:** attention, delirium, blood-brain barrier, microglia, neuroinflammation, surgery

## Abstract

Postoperative delirium is a frequent and debilitating complication, especially amongst high risk procedures such as orthopedic surgery, and its pathogenesis remains unclear. Inattention is often reported in the clinical diagnosis of delirium, however limited attempts have been made to study this cognitive domain in preclinical models. Here we implemented the 5-choice serial reaction time task (5-CSRTT) to evaluate attention in a clinically relevant mouse model following orthopedic surgery. The 5-CSRTT showed a time-dependent impairment in the number of responses made by the mice acutely after orthopedic surgery, with maximum impairment at 24 h and returning to pre-surgical performance by day 5. Similarly, the latency to the response was also delayed during this time period but returned to pre-surgical levels within several days. While correct responses decreased following surgery, the accuracy of the response (e.g., selection of the correct nose-poke) remained relatively unchanged. In a separate cohort we evaluated neuroinflammation and blood-brain barrier (BBB) dysfunction using clarified brain tissue with light-sheet microscopy. CLARITY revealed significant changes in microglial morphology and impaired astrocytic-tight junction interactions using high-resolution 3D reconstructions of the neurovascular unit. Deposition of IgG, fibrinogen, and autophagy markers (TFEB and LAMP1) were also altered in the hippocampus 24 h after surgery. Together, these results provide translational evidence for the role of peripheral surgery contributing to delirium-like behavior and disrupted neuroimmunity in adult mice.

## Introduction

Postoperative delirium is a common complication characterized by acute cognitive impairments featuring disorganized thinking, fluctuating levels of consciousness, altered arousal levels, and inattention (1). A diagnosis of delirium consistently correlates with a poor prognosis (i.e., a 5-fold increased risk for mortality at 6 months), persistent functional decline, increased nursing staff time per patient, increased hospital stays, and higher rates of nursing home placement (2, 3). This condition contributes to escalating healthcare costs previously estimated >$150 billion each year (4). Delirium is frequently seen in medical, intensive, emergency, nursing, and palliative care settings amongst any age group; however, it is the most common surgical complication in older adults (5).

Orthopedic surgery is a common intervention across every age group that can frequently contribute to cognitive impairments, emotional disturbances, and pain in a significant proportion of patients (6). As many as 50% of patients suffer from postoperative delirium after orthopedic surgery (7) and delirium is a well-appreciated predictor for mortality and adverse outcomes in patients with underlying dementia (8, 9). We have developed a mouse model of tibial fracture and repair to study the impact of orthopedic surgery on the central nervous system (CNS) and to interrogate the pathogenesis of perioperative neurocognitive disorders (10). We and others have described some of the behavioral changes affecting the hippocampus and other brain areas after surgery, although no formal evaluation of attention has been conducted to translate preclinical findings to human delirium assessment. The 5-choice serial reaction time task (5-CSRTT) is a well-established attention paradigm used to assess various aspects of attentional control in rodents, including selective, divided, and sustained attention or vigilance (11). The 5-CSRTT has been commonly used to elucidate attention impairments in several neuropsychiatric conditions including attention deficit/hyperactivity disorder (ADHD) and schizophrenia (12).

Neuroinflammation has emerged as an active driver in the pathogenesis of multiple neurological conditions, including neuropsychiatric, neurodegenerative, and perioperative disorders (13, 14). Following surgical procedures such as orthopedic surgery, pro-inflammatory cytokines have been detected in the cerebrospinal fluid of patients developing postoperative delirium (15, 16). The exact roles of these cellular processes in postoperative delirium remains elusive and their active involvement after anesthesia and surgery is generating significant interest. Recent advances in tissue clarification techniques have enabled complex 3D features in intact specimens to be observed, especially in brain (17, 18). CLARITY provides a unique approach to preserve cellular integrity through the formation of a tissue-hydrogel mesh that enables deeper optical imaging. This technology can elucidate complex 3D structures and cellular architectures of relevance to both tissue physiology and pathology, including the diagnosis of clinical conditions (19–21).

In the present study, we hypothesized that orthopedic surgery may acutely impair attention processes as assessed with the 5-CSRTT, and this delirium-like behavior may correlate with CNS inflammation assessed by microglial morphology, glial fibrillary acidic protein (GFAP) expression, and blood-brain barrier (BBB) permeability. We also implemented a CLARITY protocol combined with light sheet microscopy to enable visualization and 3D rendering of pathological features associated with postoperative complications focusing on neuroinflammation and endothelial dysfunction.

## Materials and Methods

### Animals

A total of 61 inbred C57BL6 (male, 12 weeks old) were purchased from The Jackson Laboratory (Bar Harbor, ME) and housed in the Mouse Behavioral and Neuroendocrine Analysis Core Facility. Animals were matched for weight and housed 3–5 per under controlled temperature and humidity with a 14:10 h light:dark cycle (lights on at 0600 h). Mice were fed standard rodent chow (Prolab RMH3500, Autoclavable; LabDiet, St. Louis, MO) and had access *ad libitum* access to food and water. Thirty animals were used for histological evaluation. Open field motor activity was assessed in 16 mice and was performed 24 h following fracture surgery (8 sham controls and 8 fracture animals). The remaining 15 mice were assigned to the 5-CSRTT study. One week before beginning the 5-CSRTT testing, animals were handled daily and food-restricted to achieve and be maintained at 85–90% normal body weight. Food restriction continued through the duration of the study. Once 5-CSRTT begun, mice were always fed 2–3 h following completion of training or testing. All experiments were conducted under an approved protocol from the Institutional Animal Care and Use Committee at Duke University Medical Center and under the guidelines described in the National Science Foundation “Guide for the Care and Use of Laboratory Animals” (2011). Duke University is an AAALAC certified institution.

### Surgery

Tibial facture was performed as described (10) with minor modifications. Mice were anesthetized with isoflurane using the SomnoSuite apparatus, a low-flow digital anesthesia system (Kent Scientific Corporation, Torrington, CT). Heart rate, blood oxygen saturation, and core body temperature (36.5 ± 0.6°C) were monitored throughout the procedure using pulse oximetry and a homeothermic pad system (Kent Scientific Corporation). Muscles were disassociated following an incision on the left hind-paw. A 0.38-mm stainless steel pin was inserted into the tibia intramedullary canal, followed by osteotomy, and the incision was sutured with 6-0 Prolene.

### Open Field Assessment of Activity Following Tibial Fracture Surgery

Motor activity was assessed in a 5-min open field test within 24 h of surgery for animals subjected to tibia fracture with sham controls matched for age and weight. Testing began when mice were singly placed into a 60 × 40 × 24 cm polyfoam box indirectly illuminated at 375 lux with a single camera suspended overhead interfaced to a computer running Ethovision 11.5 software (Noldus Information Technology Inc., Leesburg, VA). Activity of the mice was assessed with 3-point tracking and the total distance moved (cm) was scored for each animal.

### 5-Choice Serial Reaction Time Task (5-CSRTT)

Testing occurred in 5-CSRTT chambers (24 × 18.5 cm; MedAssociates, St. Albans, VT). Each operant chamber had five 1.24 cm^2^ nose-poke apertures, illuminated with LED lights, with infrared diodes to register nose pokes into the aperture. A single food magazine with an LED light and infrared diodes was positioned on the opposite wall. The food magazine dispersed single food rewards (20 mg chocolate Rodent Purified Diet pellets; BioServ, Flemington, NJ). After surgery, perforated plastic mesh canvas (Darice Inc., Strongsville, OH) with 0.01 cm^2^ openings were placed on bottoms of each test chamber to ensure animal mobility. Mice were trained and tested in the same chamber during 1,000 and 1,500 h. Animals were trained with a modified procedure (11, 22). For the first week of training (phase 1), all nose poke apertures were illuminated. If mouse poked into any hole, the food magazine was illuminated and a food reward dispensed. The mice had 5 s to retrieve and consume the food reward. After a 5 s inter-trial interval (ITI) the next trial began with the illumination of the nose poke apertures. This procedure was repeated until 20 rewards were earned or a maximum of 1 h had elapsed. Once mice earned 20 rewards and showed consistent performance for 3 days, the next phase was initiated. In phase 2, a single nose poke aperture was lit for a 5 s stimulus duration (SD). Animals could respond during the 5 s illuminated period or during the 5 s limited hold (LH) period which followed if the mouse didn't make an immediate selection while the aperture was lit. If the mouse selected the correct aperture during the SD or LH periods, the food magazine was illuminated and a food reward dispensed. Mice were given 5 s to retrieve the pellet. If a mouse failed to respond during the SD or LH periods or poked into an incorrect hole, the trial was immediately terminated and a 5 s time-out (TO) was initiated before proceeding to the next ITI. Mice were trained daily with the 5 s SD and 5 s LH until the animal achieved correct responses on at least 80 percent of the trials (i.e., 32 trials of 40 total trials/day) over 3-consecutive days. Of the 15 mice which began the study, 14 were capable of being trained to nose poke for food reward in phase 1 of training. Upon meeting criterion, the SD was decreased by 0.5 s each day. If the animal was successful in achieving 80% correct responses at that presentation time, the SD was reduced the following day by 0.5 s until the mouse reached a 2 s SD. In all cases, the LH was set to 5 s. When the mice exhibited at least 80% correct responses out of 40 trials for 3 consecutive days at the 2s SD, the animals were subjected to tibial fracture surgery within 24 h. Of the 14 mice that begun training with decreased SD, only 8 animals were capable of reaching the criterion for tibial fracture surgery. Surgery was done between 0700 and 1,200 h. Animals were given ~25–28 h to recover following surgery before 5-CSRTT testing and this proceeded on day 1 post-surgery between 1,300 and 1,600 h. Testing continued daily with 40 trials per day with the 2 s SD and 5 s LH for 7 days until animals reached pre-surgical performance levels.

For all phases of training and testing, task performance was assessed by three primary measures. Correct responses were defined as the number of trials in which the mice nose-poked into the correct aperture during the SD or LH divided by the total number of trials administered (40 trials) and expressed as a percentage. The accuracy rate was calculated as the total number of trials in which the mouse made a correct response divided by the number of trials in which the animal made a nose-poke response and was expressed as a percentage. Finally, the omission rate was calculated as the number of trials in which the mouse failed to respond divided by the total number of trials (40 trials) and expressed as a percentage. In addition, the latency to nose-poke (s) was recorded for each trial. On trials when a mouse failed to nose-poke, a maximum latency of 7 s was recorded (2 s SD + 5 s LH).

### Tissue Collection for Brain Samples

Under isoflurane anesthesia, mice were perfused transcardially with 30–50 mL PBS (Gibco, #10010-023), followed by 20–30 mL 4% paraformaldehyde (PFA) (#158127, Sigma-Aldrich, St. Louis, MO) in PBS, pH 7.4. Brains were harvested and post-fixed in 4% PFA at 4°C for 24 h. Brains were sliced into 1 mm thick coronal slices according to specific stereotaxic coordinates (Bregma 1.4 to 0.4; −1.2 to −2.2; −2.2 to −3.2 mm) with a vibratome. Slices were incubated in 1x PBS at 4°C for 24 h before beginning polymerization.

### Tissue Polymerization and Active Tissue Clearing

Each 1 mm thick slice was incubated in 1 mL X-CLARITY™ Hydrogel Solution (#C1310X; Logos Biosystems, Annandale, VA) at 4°C for 24 h. After incubation, polymerization was performed using the X-CLARITY™ Polymerization System (Logos Biosystems) at 37°C for 3 h. Active tissue clearing occurred using the X-CLARITY™ Tissue Clearing System (Logos Biosystems) and electrophoretic tissue clearing (ETC) solution (#C13001; Logos Biosystems) with the following setting: 0.9 A, 37°C, for 3 h. After clearing, slices were washed 3x with PBS- 0.2% Triton™-X100 (#T8787; Sigma-Aldrich) solution overnight on a shaker at room temperature.

### Tissue Labeling

After tissue clearing, each slice was incubated with primary antibody diluted in 1 mL blocking buffer (10% normal donkey serum in PBS-0.2% Triton^TM^-X100 solution) at 4°C for 3 days. Slices were washed 3x in PBS-0.1% Triton^TM^-X100 solution at 4°C overnight. Slices were incubated in secondary antibody and DAPI diluted in 1 mL blocking buffer at 4°C for 3 days. Slices were washed 3x in blocking buffer at 4°C overnight and were stored in PBS at 4°C before mounting. Details of the primary and secondary antibodies can be found in Table 1.

**Table 1:** Antibodies used for immunostaining on clarified tissues.

**Antibody**	**Company**	**Catalog#**	**Species**	**Dilution**
Iba-1	Waco	019-19741	Rabbit	1:500
GFAP	Dako	Z0334	Rabbit	1:500
Claudin 5	ThermoScientific	352588	Mouse	1:200
CD31	R&D systems	AF3628	Goat	1:200
TFEB	Invitrogen	PA5-96632	Rabbit	1:500
LAMP1	DSHB	AB528127	Mouse	1:200
AQP4	Mllipore	AB3594	Rabbit	1:500
Fibrinogen	Dako	A0080	Rabbit	1:200
Alexa Fluor Cy3 anti-Rabbit IgG (H+L)	JacksonImmuno	711-165-152	Donkey	1:200
DAPI	Sigma	D9542	–	1:1,000

### Imaging Setup

Slices were embedded with 1% agarose (#BP160-100, ThermoFisher Scientific, Waltham, MA) and placed in 1 mL syringes (#300013; BD Biosciences, San Jose, CA) without tips and incubated with mounting solution (#C13101; Logos Biosystems) at 4°C overnight followed by 1 h incubation at room temperature before imaging. Slices were imaged with a light sheet fluorescence microscope (LSFM, Z1; Zeiss, Germany) at the Duke University Light Microscopy Core Facility. During imaging, slices were immersed with mounting solution in a CLARITY optimized sample chamber. Slices were rotated to be parallel with the glass of the detection lens. All z-stacks and tile scans were acquired with 16-bit depth and 1,920 × 1,920 pixels using 5x or 20x objectives. Refractive index of the detection objective was set as 1.46. For laser excitations, the 405/488/561/640 filter set was used. Laser intensities were set between 0.5 and 2%. The exposure time for each frame was 29.97 ms. The step interval between frames was 0.659 μm.

### Image Analyses

Tile scans were stitched together and presented as high resolution 3D rendering and corresponding movies using Arivis 4D viewer (Washington, DC). The 3D rendering of single z-stack was performed with Imaris software 8.21 (Bitplane USA, Concord, MA). Immunoreactivity intensity for each channel was calculated automatically with Imaris. For automated detection of glia cells the “surface” function of Imaris was used. Volume size of each glial cell was automatically calculated after removing those segmented at the edges of the image frame. The 3D algorithm-based surface rendering and quantification of fluorescence intensity for Iba-1, IgG, GFAP, and claudin-5 were performed with Imaris at 100% rendering quality. Each channel was analyzed separately. 3D surface rendering detected immunostained cells and their processes. The channel mean intensity filter was applied and minimum thresholds were used for all the experimental groups. Voxel-based surface rendering was applied to each channel. The preset parameters remained constant throughout the subsequent analysis of Iba-1, IgG, GFAP, and claudin-5 immunoreactivities. The quantification of microglia and astrocyte number (GFAP co-labeled with DAPI) was facilitated using the Spot tools of Imaris module. All the adjustments during image processing in Imaris and Arivis were performed under identical conditions for the surgical and control groups.

### Dextran Imaging Studies to Detect Blood-Brain Barrier (BBB) Permeability

Mice were deeply anesthetized with isoflurane followed by transcardial infusion of TMR (Tetramethyl Rhodamine) 70kD dextran tracer (ThermoFischer Scientific) that was diluted in sterile PBS into 2 mM stocks and stored from light at −20°C. After 5 min, mice were perfused using 20 mL of 0.1 M PBS. The brains were harvested immediately and embedded in optimal cutting temperature (OCT) compound on dry ice (Tissue-Tek, USA). The blocks were cut into 10 μm sections using a cryostat (Microm HM550; Thermo Scientific, Waltham, MA) and the sections transferred to slides placed at room temperature. Once dried they were stored at −80°C. On the day of staining, the slides were thawed at RT for 10 min and the sections fixed with 4% PFA for 10 min at room temperature followed by washing in PBS for 5 min. Subsequently, sections were blocked using blocking buffer made of 10% normal donkey serum (#D9663; Sigma-Aldrich) with 0.3% Triton X-100 in PBS for 1 h at RT. The slides were then incubated overnight at 4°C with CD31 primary antibody (1:200, Goat CD31, R&D Systems, USA), diluted in the blocking buffer. The next day, the slides were washed with blocking buffer for 15 min followed by secondary antibody incubation in the above buffer for 1 h at room temperature. For CD31 immunostaining, donkey anti-goat Alexa 633 was used at a dilution of 1:500 (ThermoFischer Scientific). For staining of nuclei, DAPI (4,6-diamidino-2-phenylindole dihydrochloride, 1:1,000; Sigma-Aldrich) in PBS was incubated for 20 min. The sections were mounted, dehydrated and cover-slipped. Images were acquired using Zeiss 880 inverted confocal Airy scan. For the quantification, z-stack images were imported into Imaris x64 (version 9, Bitplane AG, Zurich, Switzerland) for 3D surface rendering and volumetric analysis. After display adjustment, the DAPI filter was removed to view the images in blend mode with rendering quality set to 100%. A volume filter was then applied to remove non-specific staining and minimum thresholds were used for both control and surgery groups. To quantify dextran volume, a “Filament Trace” was created from a masked surface channel of CD31. Later, by removing the CD31 filter the dextran volume was quantified using same threshold parameters for both control and surgery groups.

### Statistics

The data were expressed as mean ± S.E.M and sample sizes (*n*) are provided in the figure legends. The behavioral data were analyzed with SPSS 25 (IBM SPSS Statistics, Chicago, IL) using repeated measures ANOVA (RMANOVA) where test day was used as the within-subject variable for 5-CSRTT. For open field testing, a comparison of distance moved for surgical animals compared to shams was examined with an independent samples *t*-test. Statistical analyses for the imaging data were made using Prism GraphPad 8.0 (GraphPad Software, San Diego, CA). Bonferroni corrected pair-wise comparisons were used for *post-hoc* analyses. In all cases, a *P* < 0.05 was considered statistically significant.

## Results

### 5-CSRTT

**Figure 1:**
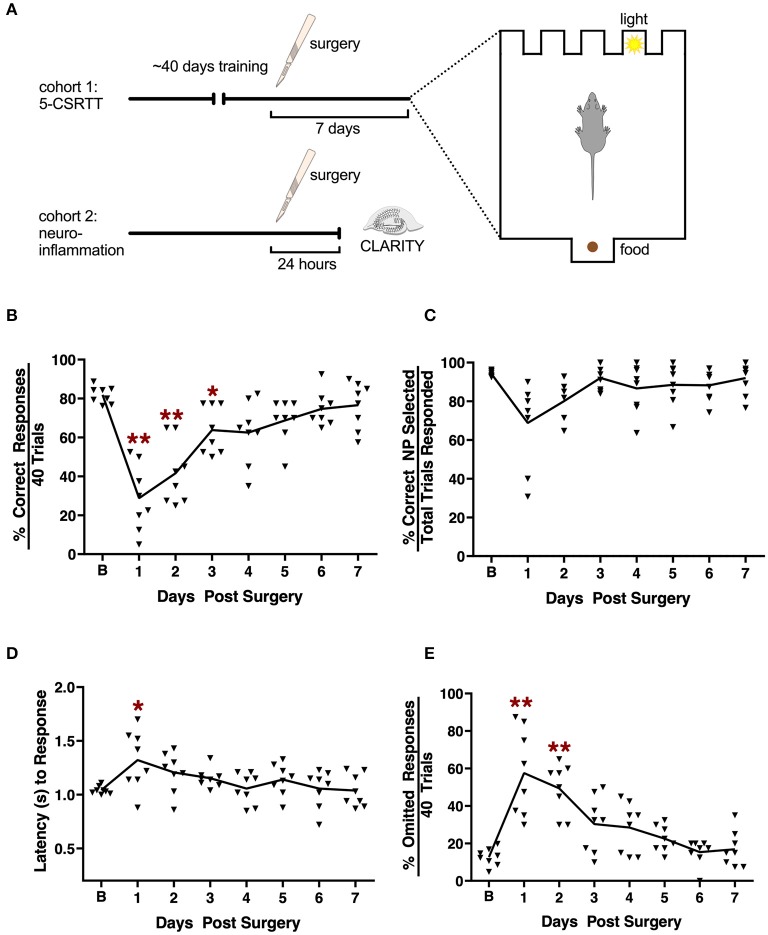
Effects of tibial fracture on attention processes using the 5-choice serial reaction time task. **(A)** Schematic of the experimental design. **(B)** Tibial fracture significantly impaired overall performance over the first 3 days post-surgery as assessed by the percent correct responses out of a total of 40 trials/day. Percent accuracy **(C)** showed small but non-significant changes following surgery. Latency (s) to correct responding **(D)** was prolonged on day 1 post-surgery compared to baseline prior to surgery. **(E)** The percent omission errors increased significantly for 2 days post-surgery before returning to baseline. Baseline 5-CSRTT performance represents the mean performance on the last 4 prior to surgery. *n* = 8 mice; results are presented as means ± SEMs; **(A–C)** ^*^p < 0.05, ^**^p < 0.01 Bonferroni *post hoc* for post-surgical time point compared to pre-surgical “baseline”.

Attention impairment is a salient component of delirium (1). Here, we assessed attention using the 5-CSRTT task before and following orthopedic surgery (Figure 1A). Several measures of performance were evaluated, including the percent correct responses, accuracy, omitted responses, and the latency for the correct responses. A RMANOVA for the percent correct responses revealed a significant effect across days [*F*_(7, 49)_ = 21.716, *p* < 0.001; Figure 1B]. Mice were significantly impaired over the first 3 days post-tibial fracture (*p* ≤ 0.043–0.001). For percent accuracy the RMANOVA revealed a significant time effect [*F*_(7, 49)_ = 4.612, *p* < 0.001; Figure 1C], however, Bonferroni corrected *post-hoc* comparisons failed to find a significant reduction in accuracy on the first day following surgery compared to baseline (*p* = 0.346). Interestingly, the mean overall latency to respond to the correct nose-poke aperture (Figure 1D) was highly significant [*F*_(7, 49)_ = 4.164, *p* < 0.001]. Despite this small, albeit significant delay across test days, the Bonferroni comparisons failed to find a significant difference between day 1 post-surgery compared to baseline (*p* = 0.599). Tibial fracture was sufficient to induce an increase in omission errors [*F*_(7, 49)_ = 16.932, *p* < 0.001; Figure 1E] over the first 2 days post-surgery. Bonferroni analyses revealed significant increases in omission errors on days 1 and 2 post-surgery compared to the baseline omission rate (*p* ≤ 0.026–0.001). As a control, we evaluated general locomotor activity 1 day after surgery and found no significant differences, including changes in body weight or food intake, that may have confounded the 5-CSRTT results (Supplementary Figure 1). Taken together, these results indicate that attention processes as evaluated with the 5-CSRTT task are significantly impaired at least on the first day post-surgery, and this deficiency is not the result of any motor impairment.

### Surgery Alters Microglial Morphology in Clarified Hippocampus

**Figure 2:**
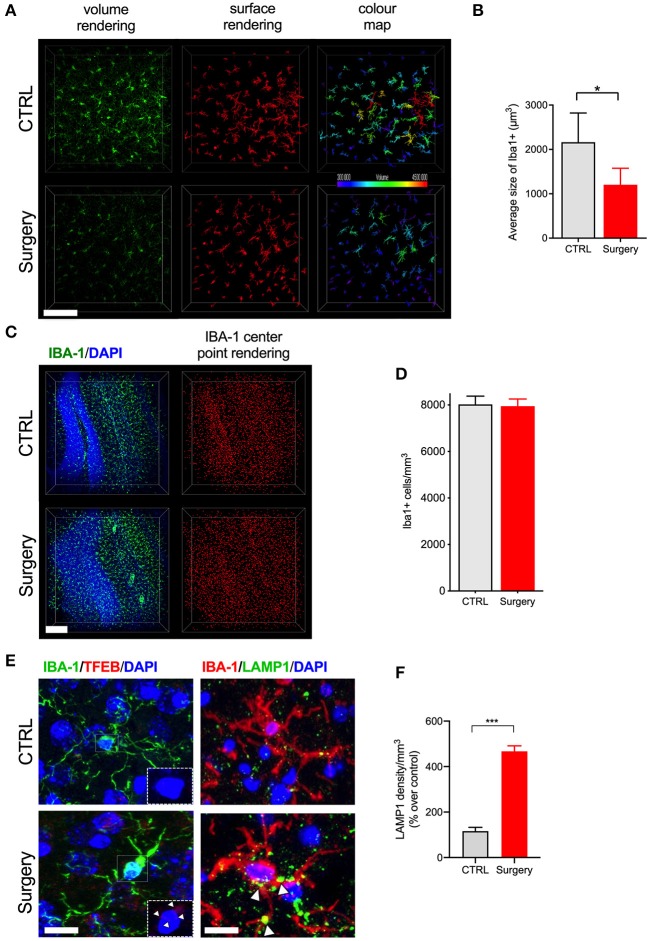
Tibial fracture alters microglial morphology and increases TFEB and LAMP1 expression. **(A)** Iba-1 staining was used to detect cytoskeletal alterations as morphological changes in microglia. Surgery significantly reduced larger, ramified, microglia. A 3D reconstruction of cell morphometry in the hippocampus is shown in the color map, with a greater loss of larger cells (green to red spectrum) in mice after tibial fracture. Scale bar: 150 μm. **(B)** Imaris-based quantification demonstrates a significant reduction in the average size of cells 24 h after surgery. *n* = 4 (CTRL) and 5 (surgery). **(C)** Iba-1 positive cell numbers were measured using center-point rendering. Scale bar: 150 μm. **(D)** Imaris-based quantification revealed no change in the Iba-1 positive cell numbers between the control and surgery groups. **(E)** Double immunostaining of Iba1 (green), TFEB (red), and Iba1 (red), LAMP1 (green) in the DG region of hippocampus. Scale bar: 2 μm. **(F)** Quantification of LAMP1 in the microglia in control and surgery groups. Tibial fracture increased the expression of both LAMP1 and TFEB puncta in the microglia. The results are presented as means ± SEMs, ^*^p < 0.05, ^***^p < 0.001 Student's *t*-test (*n* = 3 for autophagy markers).

Next we evaluated postoperative neuroinflammation using CLARITY. One mm thick brain slices were processed and cleared using electrophoresis (active clearing). Ortho-image and 3D reconstruction of the whole 1,000 μm z-stack was performed to test imaging depth and antibody penetration, and it was found to be homogeneous across clarified slices (Supplementary Figure 2, Supplementary Video 1). Orthopedic surgery activates microglial cells in the hippocampus, with morphological changes observed at 24 h post-surgery by standard histology (23, 24). Thus, we have focused our analyses at this time point using CLARITY. Surgery significantly affected microglial morphology, with an overall reduction in their ramifications (Figure 2A). Retraction of thin-ramified processes is associated with microglial activation in response to changes in the CNS microenvironment, such as pro-inflammatory cytokines and infiltrating immune cells (25). Larger, more complex cells indicative of a surveillance state, were clearly identifiable in control slices but were absent 24 h after surgery. Using surface area 3D reconstruction in Imaris we confirmed the decrease in the average volume of microglial cells, with a significant overall loss of area coverage following surgery (*p* < 0.05, Figure 2B). Further studies in surgery group failed to reveal any gross changes in the number of microglia compared to control (Figures 2C,D). Consistent with altered microglial morphology in the surgery group, we observed an increased translocation of transcription factor EB (TFEB), a master regulator of the autophagy-lysosome system along with the LAMP-1 expression that coordinates autophagy and lysosomal biogenesis (*p* < 0.001, Figures 2E,F). These results suggest that higher activity of lysosomal biogenesis in activated microglia is closely linked to the inflammation in the CNS.

### Time-Dependent BBB Opening After Surgery

**Figure 3:**
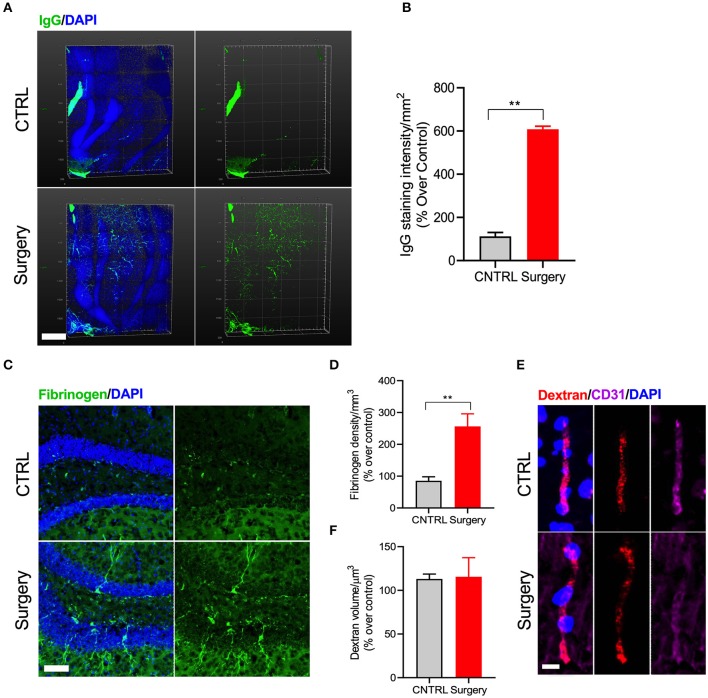
Surgery increased IgG and fibrinogen deposition. **(A)** Double staining with IgG and DAPI-nuclear stain using CLARITY. **(B)** Quantification of the IgG deposition in both control and surgery groups. Higher levels of IgG deposition are observed in the DG region of the hippocampus 24 h after orthopedic surgery. Scale bar: 500 μm. **(C)** Representative images of fibrinogen with DAPI in the DG region of the hippocampus 24 h after tibial fracture. **(D)** Quantification shows an increased fibrinogen deposition in the surgery group compared to control. Scale bar: 20 μm. **(E)** Representative images showing immunofluorescence staining for dextran tracer along with CD31 to assess brain permeability changes in mice after surgery. Scale bar: 20 μm. **(F)** Quantification of the dextran volume in the vessel. No leakage of tracer from vessels was observed, confirming that equal volume of dextran was present in both groups. The results are presented as means ± SEMs, ^**^p < 0.01 Student's *t*-test (*n* = 4).

BBB disruption is a key hallmark of many neurodegenerative conditions and it often correlates with disease progression (26). Here we used CLARITY to evaluate IgG deposition in whole hippocampal tissue 24 h after surgery (*p* < 0.001, Figures 3A,B). Surgery increased IgG deposition as compared to naive control mice (Supplementary Video 2). We confirmed these results using another marker for BBB disruption, fibrinogen, which was also up-regulated in the vessels of operated mice (*p* < 0.001, Figures 3C,D). Parenthetically, fibrinogen is a blood coagulation protein deposited in the brain during neurodegeneration that drives microglial activation (via CD11b receptor binding) and cognitive dysfunction (27). Interestingly, experiments using the 70 kD dextran tracer perfused at 24 h after surgery revealed no BBB leakage compared to control (Figures 3E,F), suggesting the barrier opens transiently within the first 24 h after surgery.

### Astrocytic and Endothelial Dysfunction After Surgery

**Figure 4:**
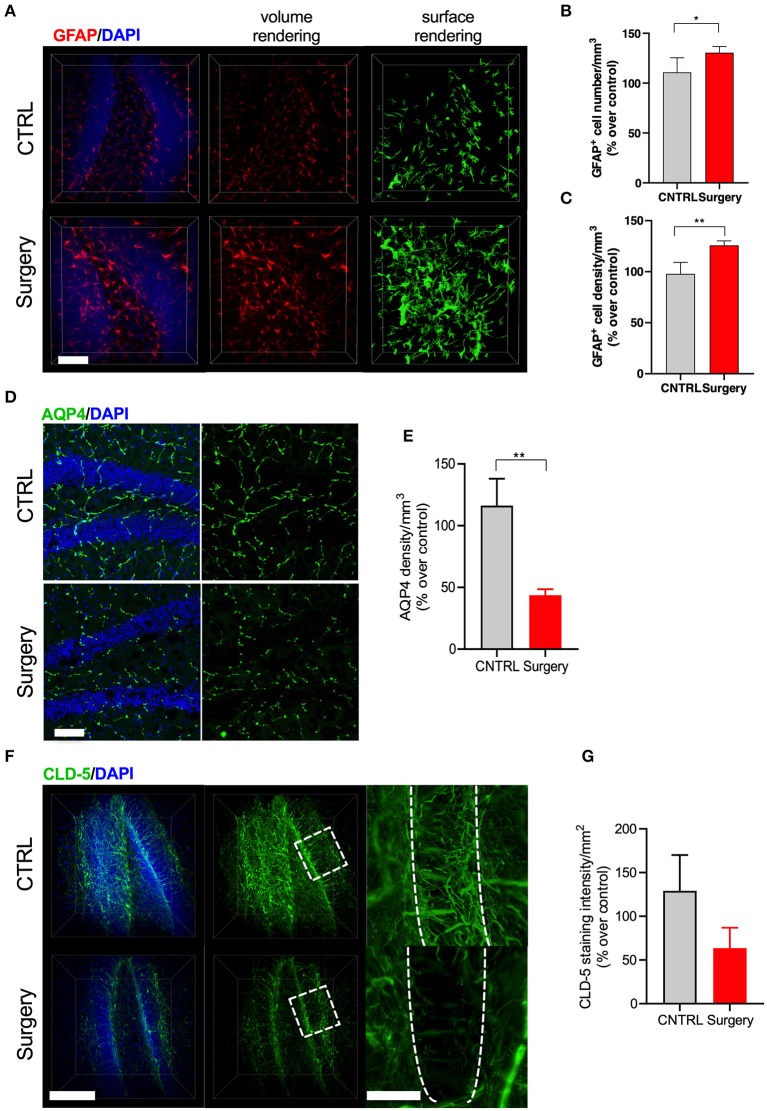
Surgery-induced astrogliosis and endothelial dysfunction. **(A)** GFAP staining and 3D reconstruction of astrocytes 24 h after surgery in clarified tissues. **(B)** Quantification revealed that surgery induces astrogliosis by modifying both cellular process and **(C)** numbers of GFAP-positive cells in the DG. **(D)** Representative image of AQP4 from the DG region of hippocampus across control and surgery groups. *N* = 4 (CTRL) and 5 (surgery). Scale bars: 20 μm. **(E)** Quantification of the percent area of AQP4 in the DG area between control and surgery groups. **(F)** 3D surface and volume rendered reconstruction of tight junction marker claudin-5 staining 24 h after surgery. *N* = 2 for CLD-5. Scale bars: 50 and 20 μm (insets). **(G)** Quantification revealed that control mice show increased CLD-5 expression while surgery significantly reduced it. The results are presented as means ± SEMs, ^*^p < 0.05, ^**^p < 0.01 Student's *t*-test.

Astrocytes are critical to the formation and maintenance of the BBB (28). At 24 h after surgery we found that orthopedic surgery induced significant changes in the expression of the intermediate filament GFAP in clarified hippocampal slices (Figure 4A). We evaluated this increase by using surface rendering in Imaris, which revealed changes both in the number of GFAP-positive astrocytes (*p* < 0.05, Figure 4B) as well as coverage area of these enlarged cells (*p* < 0.001, Figure 4C). Next, we focused on the expression of aquaporin-4 (AQP4), a water channel that is highly localized in the end feet of astrocytes that are in contact with BBB. Alterations in the expression of AQP4 has been linked to neurodegenerative diseases (29). AQP4 levels were reduced in the hippocampus of surgical mice compared to naïve controls (*p* < 0.001, Figures 4D,E). Finally, using CLARITY we measured alterations in claudin-5 (cld-5), one of several intracellular tight junction proteins that form BBB (Figures 4F,G). Surgery caused a significant disruption of cld-5 compared to control 24 h after surgery. Interestingly, the loss of cld-5 coverage was especially notable in the granular cell layer, as well as other areas of the hippocampus. Collectively, these results suggest that orthopedic surgery triggers astrogliosis and endothelial impairments in the hippocampus.

## Discussion

This study provides new evidence for the impact of peripheral surgery on a cognitive domain of major relevance to postoperative delirium. We also explored the contribution of postoperative neuroinflammation and endothelial dysfunction using CLARITY in this clinically-relevant model of orthopedic surgery.

Inattention is one of the core features of delirium and a mandatory component for diagnosis in compliance with DSM-5 criteria (30). However, delirium is a very heterogeneous syndrome characterized by multiple features and subtypes, which often make its characterization challenging and under diagnosed in hospital settings (31). Other cardinal features of delirium include an acute onset with fluctuating course, disorganized thinking, and altered level of consciousness, which can be difficult to define both in humans and preclinical models. In rodents, we and others have previously reported on different behavioral deficits affected by orthopedic surgery. These include changes in fear conditioning (23, 24, 32, 33), novel object recognition memory/open field (33, 34), zero maze (35), Y-maze (32, 36), and Morris water maze (37). While spatial and working memory tasks have overlapping mechanisms with attention (38), the 5-CSRTT allowed us to examine and manipulate more discrete aspects of attention. The 5-CSRTT is an appetitive task and is one of the most robust assays to study visuospatial attention in rodents, which is analogous the commonly used continuous performance task used measure of attention in both clinical and research settings (39). Our results using the 5-CSRTT after surgery demonstrate the most profound impairment occurs 24 h after surgery with full recovery by day 5 post-procedure. This time-course is very similar to the clinical manifestation of delirium and it provides a valid endpoint to test possible interventions. Notably, discrete aspects of 5-CSRTT performance are centrally mediated by cholinergic and other transmitter manipulations (40). Acetylcholine (ACh) has been strongly linked to selective attention processing across species, including humans (41), and cholinergic dysfunction has been proposed as a key feature of delirium (42, 43). Recent data from elective surgical patients suggest that acetylcholinesterase activity, the enzyme responsible for degrading ACh, is higher in patients with delirium compared to non-delirious controls, supporting the cholinergic hypothesis for postoperative delirium (44). Similar findings in rodents suggest that cholinergic depletion predisposes to acute cognitive impairments and microgliosis after systemic LPS challenge (45). Here, we described changes in the expression of autophagy markers in microglia, which may relate to the systemic inflammatory response and monocytic infiltration following orthopedic surgery (46–48). Whether autophagy is induced by this peripheral response or centrally as a protective mechanism for microglia to resolve neuroinflammation needs further elucidation. Gliosis and endothelial dysfunction have been recently observed in critically ill patients with delirium (49). Using CLARITY we have implemented a staining protocol to improve quantification of these changes from standard immunofluorescence and are now better able to evaluate morphological changes in these highly complex structures. Vascular patterns of BBB disruption can be observed in the hippocampus at 24 h. Interestingly, injection of 70 kDa dextran at this time point revealed no significant parenchymal leak, suggesting the barrier opens transiently. These changes can be detected using other markers, such as IgG and fibrinogen. This is consistent with other work where laparotomy triggered changes in selective tight junctions, including claudin-1, occluding, ZO-1, and 10 kDa dextran but not 70 kDa (50). Thus, it is possible for peripheral cytokines to enter the CNS and trigger delirium-like behaviors after surgery. Circulating plasma proteins, including pro-inflammatory makers like C-reactive protein and IL-6, have been recently correlated with patients developing delirium after non-cardiac surgery (51). Importantly, we previously found IL-6 to be upregulated both in the periphery as well as in the CNS (23), and administration of a selective monoclonal antibody was shown to prevent postoperative neuroinflammation and cognitive decline in this model (52). Selective blockade of pro-inflammatory cytokines may have translational implication for delirium treatment, although side effects associated with excessive immunosuppression and/or impaired wound healing will need to be evaluated carefully. Since, pro-inflammatory cytokines are detected in the cerebrospinal fluid (CSF) of patients with delirium (15, 53), blocking these mediators in the CSF without affecting the peripheral immune response may reveal alternative approaches to prevent surgery-induced neuroinflammation. Finally, many of these cytokines are already upregulated at baseline in patients at-risk for delirium. In fact, aging and chronic stress elevates IL-6 amongst other mediators (54, 55) and these cytokines can further prime microglia cells and possibly impair neuro-immune circuits of relevance to delirium-like behavior. Indeed surgery also triggers acute plasma cortisol, which was found elevated in patients with delirium after cardiac surgery (56). However, recent trials using steroids have not shown significant effects on delirium outcomes after surgery and critical illness (57, 58). In this context, delirium may be considered a surrogate marker for neuroinflammation in *selective* brain areas that may contribute to the different clinical manifestations of this disorder (59), thus requiring more selective disease-modifying therapies to treat and possibly prevent.

Preclinical models to study postoperative delirium have been notably limited. Systemic infective challenges (e.g., lipopolysaccharide or LPS) have been used to examine certain aspects of behavioral change. Culley et al. (60) have evaluated the effects of LPS using the attention set-shifting task (AST) and found selective impairments in attention/executive function in aged rats for up to 72 h post-surgery. Parenthetically, AST involves a series of discriminations based on stimulus dimensions such as digging medium or shape cue in a task analogous to the Wisconsin Card Sorting Task. Without affecting initial discrimination learning, LPS impaired reversal-learning at 24 but not at 48 h and extra-dimensional shift discrimination at 72 h. Transient changes in working memory have been similarly described in other delirium models of neurodegeneration after LPS exposure using the Y maze, T maze alternation tasks, and novel object recognition memory (61). Peng et al. (62) have evaluated certain aspects of delirium in a mouse model of abdominal surgery using composite Z scores from a battery of tests including the buried food test, open field test and Y maze. They found that anesthesia and surgery impaired both natural and learned behavior of the mice with acute onset and fluctuating course, modeling one of the cardinal features of delirium. Further, other studies have described behavioral changes after anesthesia exposures that can affect neuronal circuits related to recognition memory, arousal, and motoric behaviors (63, 64). In this study we have not included an anesthesia exposure group for several reasons. First, we previously reported that isoflurane exposure in this exact model does not trigger significant neuroinflammation, or behavioral deficits (23). We have confirmed this by assessing sham and surgical mice in the open field without observing significant deficits. Second, recent work by Hambrecht-Wiedbusch et al. showed no effects of general anesthesia (including ketamine exposure) focusing on attention in healthy rats using the 5-CSRTT (65). Last, we allowed the mice to have a full 24 h recovery before resuming testing 25 h later in order to avoid any possible effects of isoflurane exposure on food intake or nausea; although studies have shown that a single exposure of isoflurane in C57BL/6 mice does not have significant effects on food intake for up to 9 days post exposure (66).

This study presents limitations and challenges to this emerging field of preclinical research. Inattention is one of many features of delirium; in fact, its fluctuating course was not addressed by these experiments and may require other correlates, such as electrophysiology, to fully ascertain in rodents. Further, not all patients experience delirium. However, our data indicate that mice have different degrees of attentional impairment so this may provide opportunities to identify resiliency traits that may impact on perioperative cognitive outcomes. Finally, we have not addressed the contribution of advanced age and other risk factors into this model, which will be objective of future work as well as more mechanistic experiments to further identify the contribution of neuroinflammation and endothelial dysfunction as key drivers of delirium pathogenesis. Defining more specific targets at the neurovascular unit may provide much needed therapies to resolve neuroinflammation and inform future clinical trials.

In conclusion we have provided translational evidence using a well-established surgical model to study attention processes and features relevant to the pathophysiology of postoperative delirium. The utility of these endpoints can be translated into trials evaluating biomarkers of neuro-glia and endothelial dysfunction after surgery, which may contribute to a better understanding on the genesis of postoperative delirium.

## Data Availability Statement

The raw data supporting the conclusions of this manuscript will be made available by the authors, without undue reservation, to any qualified researcher.

## Author Contributions

CX optimized the CLARITY protocol. RV, CX, and SS analyzed light-sheet images. RV performed dextran experiments and immunostainings. FP and RR conducted and analyzed behavioral experiments. RR, WW, and NT designed research and provided new analytical tools. NT drafted the manuscript. All authors read and approved the final manuscript.

### Conflict of Interest

The authors declare that the research was conducted in the absence of any commercial or financial relationships that could be construed as a potential conflict of interest.

## Supplementary Material

Supplementary Figure 1 Schema for 5-choice serial reaction time task at the time of tibial fracture. **(A)** A trial is initiated once a mouse is placed into a darkened 5-CSRTT chamber. Testing begins when one of the five nose-poke apertures is randomly illuminated. The nose poke aperture is lit for 2 s and the mouse has 5 s to respond after the light is extinguished. If the mouse responds with a nose-poke in the lit aperture (“correct response”), the food magazine is immediately illuminated and a 20 mg chocolate food pellet (“reward”) is dispensed. The mouse has 5 s to consume the food reward, followed by a 5 s inter-trial interval (ITI) before the commencing of the next trial. If the mouse responds with a nose-poke into an unlit aperture (“incorrect response”) or fails to respond at all (“omission”), the mouse is given a 5 s time-out (TO), where all lights are extinguished, and the 5 s ITI. **(B)** Open field activity in mice 24-h following tibial fracture. No significant differences were found between sham (*n* = 10) and mice subjected to tibia fracture (*n* = 10) for distance (cm) traveled during a 5 min open field tested 24 h post-surgery. **(C,D)** represent body weight and food intake in the mice exposed to the 5-CSRTT task. No significant changes were observed throughout the duration of the study. Results are presented as means ± S.E.M.

Supplementary Figure 2 Schema of the clearing process and transparency of 1 mm coronal brain slices. **(A)** The transparency and agar mount for 1 mm mouse brain slice after tissue clearing. **(B)** Imaging depth was verified using Iba-1 (green)/DAPI (blue) immunostaining and **(C)** staining quality evaluated at every 200 μm z-stack intervals up to 1,000 μm (20X objective, stack size 1 mm). *n* = 5 slices. Scale bars: 4 mm **(A)**, 200 μm **(B)**, 100 μm **(C)**.

Supplementary Video 1 Iba1+ microglia in the hippocampus. Cleared brain slices were stained with Iba1 (Green) and DAPI (Blue). The video was generated using Arivis 4D viewer.

Supplementary Video 2 IgG deposition in the hippocampus 24 h after surgery. Endothelial permeability was assessed by IgG staining (Green) followed by DAPI counterstain (Blue). The video was generated using Arivis 4D viewer.
