# Supplementary figures and images for: Orthopedic Surgery Triggers Attention Deficits in a Delirium-Like Mouse Model
Source: Front Immunol. 2019 Nov 19;10:2675. doi: 10.3389/fimmu.2019.02675 (PMC6918861; doi:10.3389/fimmu.2019.02675)

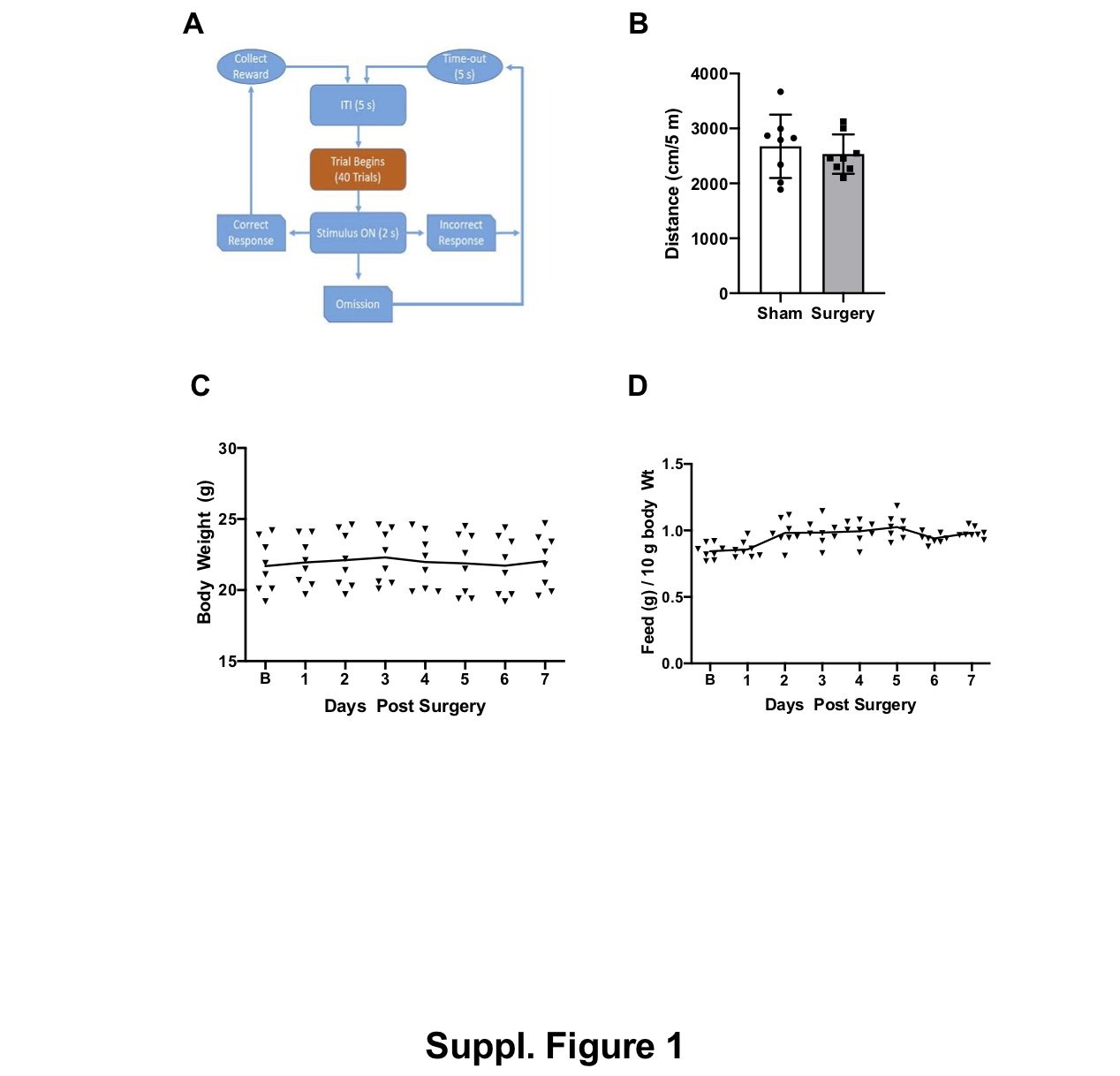

Supplement: Supplementary Figure 1 — Schema for 5-choice serial reaction time task at the time of tibial fracture. (A) A trial is initiated once a mouse is placed into a darkened 5-CSRTT chamber. Testing begins when one of the five nose-poke apertures is randomly illuminated. The nose poke aperture is lit for 2 s and the mouse has 5 s to respond after the light is extinguished. If the mouse responds with a nose-poke in the lit aperture (“correct response”), the food magazine is immediately illuminated and a 20 mg chocolate food pellet (“reward”) is dispensed. The mouse has 5 s to consume the food reward, followed by a 5 s inter-trial interval (ITI) before the commencing of the next trial. If the mouse responds with a nose-poke into an unlit aperture (“incorrect response”) or fails to respond at all (“omission”), the mouse is given a 5 s time-out (TO), where all lights are extinguished, and the 5 s ITI. (B) Open field activity in mice 24-h following tibial fracture. No significant differences were found between sham (n = 10) and mice subjected to tibia fracture (n = 10) for distance (cm) traveled during a 5 min open field tested 24 h post-surgery. (C,D) represent body weight and food intake in the mice exposed to the 5-CSRTT task. No significant changes were observed throughout the duration of the study. Results are presented as means ± S.E.M. [file Image_1.TIFF]

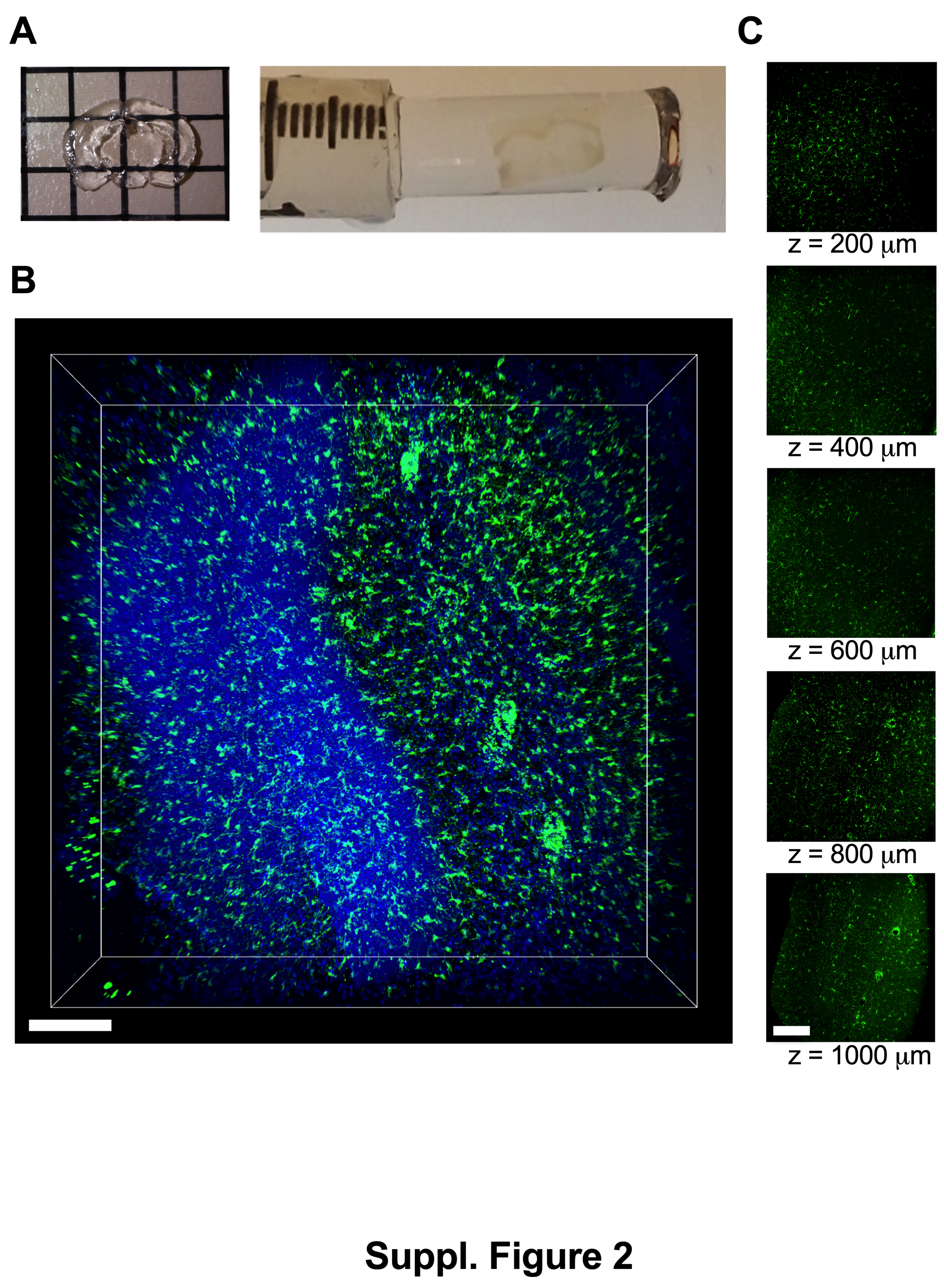

Supplement: Supplementary Figure 2 — Schema of the clearing process and transparency of 1 mm coronal brain slices. (A) The transparency and agar mount for 1 mm mouse brain slice after tissue clearing. (B) Imaging depth was verified using Iba-1 (green)/DAPI (blue) immunostaining and (C) staining quality evaluated at every 200 μm z-stack intervals up to 1,000 μm (20X objective, stack size 1 mm). n = 5 slices. Scale bars: 4 mm (A), 200 μm (B), 100 μm (C). [file Image_2.TIFF]
